# Effects of traditional Chinese exercises on mental health in individuals with drug rehabilitee: A systematic review and meta-analysis

**DOI:** 10.3389/fpubh.2022.944636

**Published:** 2022-08-04

**Authors:** Yulong Zhang, Shenghua Lu

**Affiliations:** ^1^Hunan Judicial Police Vocational College, Changsha, China; ^2^College of Sports Science, Jishou University, Jishou, China; ^3^Hunan Academy of Education Sciences, Changsha, China

**Keywords:** Tai Chi, Qigong, depression, anxiety, drug addiction

## Abstract

**Purpose:**

The intent of this systematic review and meta-analysis was to examine the effects of Traditional Chinese Exercise (TCE) on mental health and drug cravings in drug rehabilitees.

**Methods:**

Six electronic databases (PubMed, Web of Science, MEDLINE, CINAHL, PsycArticles, and CNKI) were searched to identify the potential literature from inception to March 2022. The controlled studies with a pro-posttest design that investigated the effects of TCE on mental health (depression, anxiety, drug craving, and sleep quality) were included. The effect sizes were calculated using the random-effect models with a 95% confidence interval. The Physiotherapy Evidence Database (PEDro) scale was employed to evaluate study quality.

**Results:**

A total of 10 studies (740 participants, mean age 35 years old) were included in this study. The pooled results showed that TCE produced significant improvements in depression (SMD = 0.65, 95% CI 0.29 to 1.02, *p* < 0.01), anxiety (SMD = 0.98, 95% CI 0.44 to 1.53, *p* < 0.01), and drug craving (SMD = 0.87, 95% CI 0.54 to 1.21, *p* < 0.01) compared to the control group. The subgroup analysis results showed that TCE resulted in significant improvements in depression compared to active intervention (SMD = 0.33, 95% CI 0.07 to 0.60) or passive intervention (SMD = 1.07, 95% CI 0.40 to 1.74). A significant improvement in depression was observed in both male and female drug rehabilitee (*p* < 0.05). Moreover, Tai Chi (SMD = 0.69, 95% CI 0.19 to 1.18) or Qigong (SMD = 0.49, 95% CI 0.24 to 0.74) exercise, 3–4 times per week (SMD = 1.06, 95% CI 0.39 to 1.74) or ≥5 times (SMD = 0.39, 95% CI 0.12 to 0.66), >45 min (SMD = 0.62, 95% CI 0.09 to 1.15) or ≤ 45 min (SMD = 0.68, 95% CI 0.10 to 1.27), and for a duration of 12 weeks (SMD = 0.84, 95% CI 0.15 to 1.54) produced significant improvement in depression.

**Conclusion:**

This current study suggests that TCE (Tai Chi, Qigong) may have benefits in alleviating depression, anxiety, and drug cravings in drug rehabilitees. Further studies are required to verify our results through the implementation of well-designed experimental protocols.

## Introduction

Drug addiction (also known as substance use disorder) is a progressive and elapsing mental illness that causes individuals to lose control of substance abuse despite its associated detrimental (life-threatening) effects (1). Specifically, drug addiction has been widely accepted as being associated with significant changes in brain structure and function, resulting in the desensitization of drugs increasing dosage is needed to help drug users produce the same effect (1). The global prevalence of illicit drug use has reached 5.2% in 2015 (2). This number has increased in some countries/regions; for example, authoritative data have indicated that roughly 15.3% of US citizens have been diagnosed with drug addiction or dependence (3, 4). Similarly, in another country, Australia, this number (illicit drug use) has reached 13% among individuals aged 14 and greater (5). Compared with these above-mentioned countries, Yunan has been widely recognized as one of the provinces most affected by drug abuse in China and its estimated prevalence (registered data of illicit drug users) was almost 1% in spite of the standardized punishment policy (6).

The detrimental consequences of this mental illness are linked to a greater susceptibility of being affected by HIV/AIDS and hepatitis C, a high crime rate, and the development of other co-existing chronic illnesses, such as cardiovascular disease (7). In addition, mental health problems (anxiety, depression, and insomnia) are commonly reported among drug users (8). This comorbidity of mental illnesses contributes to a reduced quality of life, disability, and premature death (9, 10). Economic consequences of drug abuse involve immeasurable harm to public health and safety worldwide, with a global cost of about USD 35 billion per year (11). These financial burdens, to a large extent, have challenged the healthcare system in countries of different economic levels. Direct costs and hospitalization are not affordable for families with drug users, especially in developing and economically disadvantaged countries and regions. Taken together, drug users should receive greater attention from the research community and clinicians.

Pharmacological treatments for drug users have mainly focused on specific receptors within the dopaminergic system, glutamate system, and trace amine system, and they can target the addiction-related systems in the brain to reduce the level of withdrawal and other symptoms (1, 12). Of note, a drug's side effects on the physical and psychological health of users should be pointed out as well. To this end, non-pharmacological treatments have been developed, such as cognitive-behavioral therapy, contingency management, residential rehabilitation based therapies, and repetitive transcranial magnetic stimulation (13). Besides these approaches, exercise as a cost-effective treatment has been increasingly used to alleviate symptoms related to drug addiction (14, 15). A previous review published in 2014 has reported the beneficial effects of physical exercise for drug users (16). With an increasing number of studies on this topic in the last 10 years, an updated review is urgently needed. In addition, this meta-analysis did not focus on a unique exercise modality alone - traditional mindful exercise. Traditional Chinese exercises (TCEs) include Tai Chi and Qigong, which have attracted an increasing number of scholars investigating their benefits on populations (e.g., reducing one's risk of falls and improving mental health). TCE is characterized by slow movements, a deep breathing technique, and meditation training and these unique elements can help drug users alleviate negative emotions caused by withdrawal. Ultimately, the exercises assist one in achieving symptomatic management and obtaining a better quality of life. Indeed, earlier studies have suggested that TCE contributes to decreasing the symptoms of depression and anxiety in adults with or without diseases, such as healthy adults (17) and adults with major depressive disorder (18) or chronic obstructive pulmonary disease (19). Furthermore, individuals who practice TCE might be able to improve their wellbeing (20).

Although previous studies have investigated the beneficial effects of TCE on mental health in different populations, few systematic review studies have investigated the effects of TCE on mental health in subjects suffering from substance use disorders (21, 22). Additionally, two studies reported contradictory results. One study found that Tai Chi had a significant effect in reducing depression (21), while another study found the opposite (22). Moreover, the inclusion criteria of both reviews were not very strict. For example, the participants in Zhang et al.'s study included drug users and alcohol abusers. Different types of intervention (e.g., Qigong + medication) were included in the experimental group (21). Moreover, the quality of included studies was low because of the nature of non-randomized controlled studies. For example, the review study of Liu et al. included one randomized controlled study and six non-randomized controlled studies (22). It also did not investigate the effects of the dose of intervention on mental health in drug rehabilitee due to the limited number of included studies.

Therefore, based on the limited information of these systematic reviews, an updated meta-analysis on these unique exercise modalities should receive greater attention, and the results could be used to help clinicians treat symptoms and drug users if the exercises are practiced on a regular basis.

## Methods

### Search strategy

We searched for publications in six electronic databases (PubMed, Web of Science, MEDLINE, CINAHL, PsycArticles, and CNKI) from their inception until March 2022. The following terms were combined to retrieve potential studies: (1) “Tai Chi” OR “Taijiquan” OR “Qigong” OR “Baduanjin” OR “Wuqingxi” OR “Dao Yin”; (2) “Amphetamine” OR “Cocaine” OR “Heroin” OR “Morphine” OR “Methadone” OR “Cannabis” OR “Drug Abuse” OR “Drug Dependence” OR “Substance Abuse”; (3) “depression” OR “anxiety” OR “sleep^*^” OR “drug craving”. In addition, manually search for bibliographies and systematic review studies were performed to identify relevant studies. The current meta-analysis study was followed by the PRISMA (Preferred Repointing Items for Systematic Reviews and Meta-Analyses) (23).

### Inclusion and exclusion criteria

Studies were included if they met the following criteria: (i) participants had a drug addiction; (ii) participants only practiced Tai Chi, Qigong, or other types of TCE intervention in the experimental group, and did not use TCE interventions in the control group; (iii) the study reported at least one outcome measurement (i.e., depression, anxiety, and sleep). (v) the study was published in English or Chinese. The exclusion criteria were: (i) other types of studies (e.g., animal or *in vitro* studies, abstracts, case reports, reviews); (ii) the control group included Tai Chi or Qigong exercise; (iii) the intervention duration was not no less than 8 weeks; (iv) there was insufficient information to calculate the effect size of the outcome measurement.

### Data extraction and quality assessment

Two authors independently assessed the eligible studies by screening the titles, abstracts, and full texts. A mutual communication was conducted when two authors had disagreements on inclusion or exclusion criteria. The included articles were analyzed following the predesignated structure: author, year of publication, characteristics of subjects, sample size, sex, age, intervention protocol (frequency, time, type, duration, and intensity), outcome measurements (depression, anxiety, drug craving and sleep).

The quality of included studies was evaluated using the Physiotherapy Evidence Database (PEDro) scale (24) by two independent reviewers. This PEDro scale is a systematic tool with adequate content to assess the study quality that includes 11 items, of which Item 1 is not involved in calculating the total scores. The total scores ranged from 0 to 10. The study quality was classified as excellent (9–10 points), good (6–8 points), fair (4–5 points), and poor (<4 points) (24).

### Statistical analysis

Comprehensive Meta-Analysis software v3 software (Biostat, NJ) was employed for data analyses in this meta-analysis study. The standardized mean difference (SMD) was expressed as the effect size (ES), with a corresponding 95% confidence interval (CI) and *p* value (25). The SMD of each study was calculated by comparing the mean change from pre- to post-intervention in the experimental and control groups. The pooled ESs were determined using a randomized effect model and shown in a forest plot, and a positive ES value expressed a positive effect of TCE. The ES was commonly defined by three layers: small (0.2–0.49), moderate (0.50–0.79), and large (≥0.8) (26). Meanwhile, the *I*^2^ statistic test was used to examine heterogeneity across studies. *I*^2^ <25%, *I*^2^ <50%, and *I*^2^ <70% were considered as low heterogeneity, moderate heterogeneity and high heterogeneity, respectively (27). Publication bias was examined using the funnel plot and Egger's test, and it was not included if the number of included studies was less than 10. Sensitivity analysis was conducted to assess the stability of the results by excluding one study each time. Furthermore, subgroup analysis was performed to explore sources of heterogeneity by the dose of exercise: exercise type - Tai Chi and Qigong; exercise frequency - 1 to 2 times, 3 to 4 times, and more than 5 times per week; exercise duration- 12 weeks, 13–23 weeks, and 24 weeks or more; exercise session time – 30 to 45 min, and more than 45 min (28). The control group was divided into active group (i.e., recreation activity) and passive group (i.e., no treatment). The significance level was set at *p* < 0.05.

## Results

### Search results

**Figure 1:**
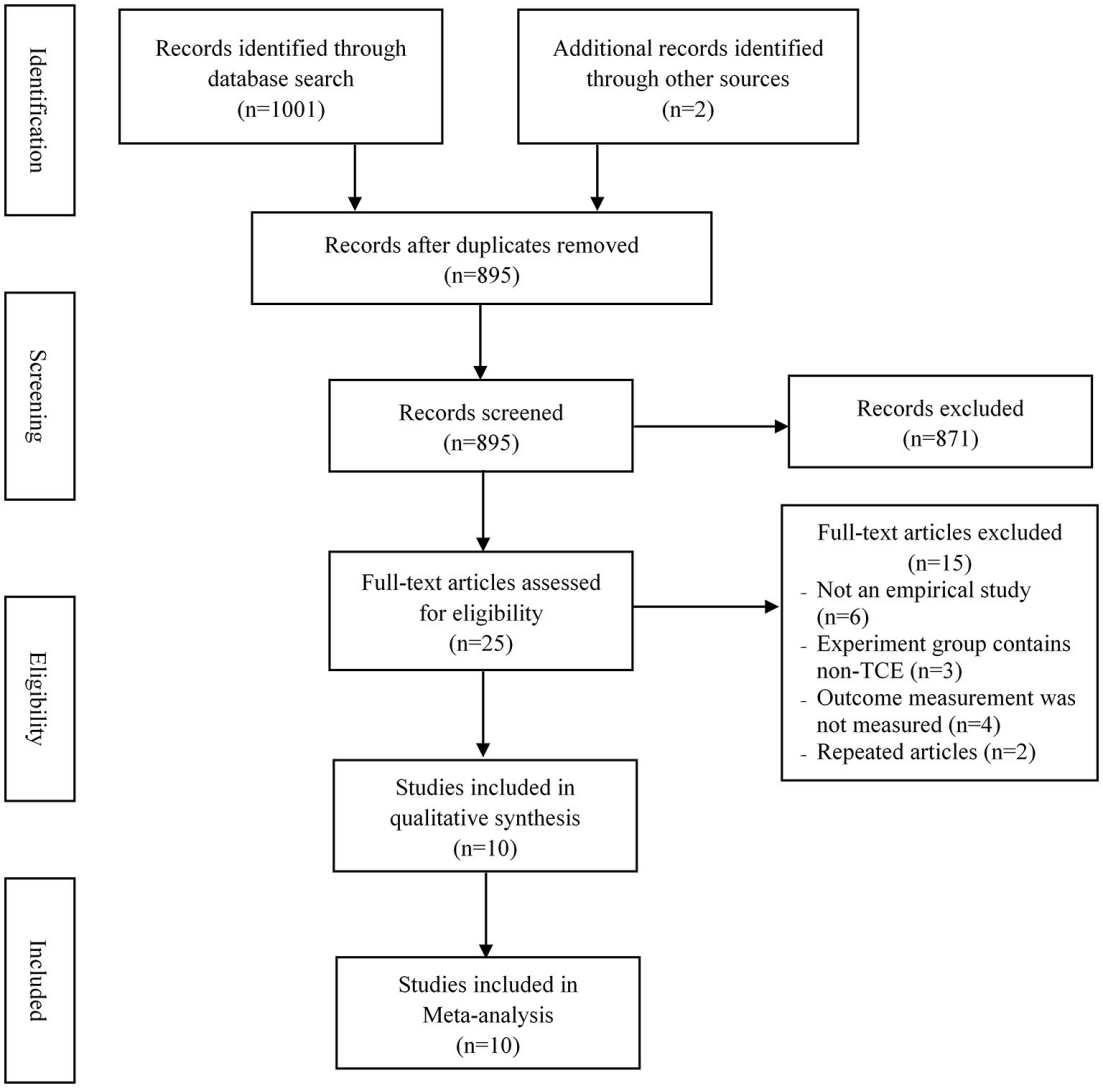
The flowchart of the study selection process.

The PRISMA flowchart (Figure 1) presents the study selection in the meta-analysis study. A total of 1,003 studies were retrieved from the database. After removing duplicates (*n* = 108), 895 studies were reviewed and 25 studies remained. Subsequently, 15 studies were excluded based on the reasons shown in the flowchart. Finally, 10 studies (29–38) were selected for inclusion in this meta-analysis.

### Study characteristics

Table 1 presents the characteristics of the included studies. These studies were mainly conducted in China, with a total of 740 participants. Three studies were published in the English language (35, 36, 38). The mean age of participants was 35 years old. For TCE intervention, 8 studies used Tai Chi and 2 studies used Qigong exercise. The exercise session of the included studies ranged from 30 min to 80 min, the exercise frequency was 2 to 7 times per week, and the exercise intervention lasted between 12 and 24 weeks. With respect to outcomes, depression, anxiety, drug cravings, and sleep were measured in 10 studies (29–38), 8 studies (29–34, 37, 38), 3 studies (34, 35, 38), and 3 studies (32, 36, 37), respectively. Additionally, with regard to outcome measurements, depression was measured using the self-rating depression scale (SDS) by Zung, the Baker self-rating depression scale (BDI), and the Hamilton Rating Scale for Depression (HRSD); anxiety was measured using the self-rating anxiety scale (SAS) by Zung; drug cravings were measured using the desire for speed questionnaire by Wang (39); and the Pittsburgh Sleep Quality Index (PSQI) was used to measure sleep quality.

**Table 1:** Characteristics of the included studies.

**References**	**Mean age (years) Sample size**	**Gender**	**Time/Frequency/ Duration**	**Interventions**		**Instrument/ Outcomes**	
				**Experiment (Details)**	**Control**		**Findings**
Sui (33)	30 E = 28; C = 30	Male	60min/3times/week 16 weeks	Qigong	No treatment	SDS/depression SAS/anxiety	The Qigong exercise had a significant effect in reducing the depression and anxiety symptoms compared to the control group.
Li et al. (35)	30 E = 17; C = 16	Female	80min/2times/week 24 weeks	Tai Chi	Recreation activity	HRSD/depression HWS/drug craving	There were no significant differences in depression and drug craving between Tai Chi and control groups.
Geng et al. (29)	36 E = 30; C = 27	Female	45min/5tims/week 12 weeks	Tai Chi	Recreation activity	SDS/depression SAS/anxiety	The Tai Chi exercise had a significant effect in reducing the depression and anxiety symptoms compared to the control group.
Zhang and Zhu (38)	40 E = 38; C = 34	Male	50min/5times/week 24 weeks	Tai Chi	Recreation activity	BDI/depression SAS/anxiety DSQ/drug craving	The Tai Chi exercise had a significant effect in reducing the depression, anxiety and drug craving symptoms compared to the control group.
Wang (34)	38 E = 24; C = 25	Female	30min/6times/week 12 weeks	Tai Chi	Recreation activity	SDS/depression SAS/anxiety DSQ/drug craving	The Tai Chi exercise had a significant effect in reducing the depression, anxiety and drug craving symptoms compared to the control group.
Zhao (32)	39 E = 35; C = 37	Male	70min/3times/week 12 week	Tai Chi	No treatment	SDS/depression SAS/anxiety PSQI/sleep quality	The Tai Chi exercise had a significant effect in reducing the depression, anxiety and improving sleep quality compared to the control group.
Zheng (31)	36.5 E = 25; C = 27	Male	60min/5times/week 12 weeks	Tai Chi	Recreation activity	SDS/depression SAS/anxiety	The Tai Chi exercise had a significant effect in reducing the depression and anxiety symptoms compared to the control group.
Zhu et al. (36)	36 E = 42; C = 38	Female	60min/3-5times/week 24 weeks	Tai Chi	Recreation activity	SDS/depression PSQI/sleep quality	The Tai Chi exercise had a significant effect in reducing the depression and improving sleep quality compared to the control group.
Fu et al. (37)	28 E = 100; C = 100	Female	30min/7times/week 20 weeks	Qigong	No treatment	SDS/depression SAS/anxiety PSQI/sleep quality	The Qigong exercise had a significant effect in reducing the depression, anxiety and improving sleep quality compared to the control group.
Zhu (30)	30 E = 50; C = 50	Male	60min/3tims/week 15 weeks	Tai Chi	No treatment	SDS/depression SAS/anxiety	The Tai Chi exercise had a significant effect in reducing the depression and anxiety symptoms compared to the control group.

*BDI, Baker self-rating depression scale; C, Control group; DSQ, Desire for speed questionnaire; E, Experimental group; HRSD, Hamilton Rating Scale for Depression; HWS, Rating Scale of Heroin Withdrawal Symptoms; SAS, Self-rating anxiety; SDS, Self-rating depression; PSQI, Pittsburgh Sleep Quality Index*.

The study quality assessment was used the PEDro scale (Table 2). The study quality scores ranged from 5 to 8 points, indicating that the included studies were considered as fair and had good methodological qualities. Only three studies used a blinding method to measure the outcomes (32, 36, 38).

**Table 2:** Methodological quality of the included studies.

**References**	**Score**	**Methodological quality**	**PEDro item number**										
			**1**	**2**	**3**	**4**	**5**	**6**	**7**	**8**	**9**	**10**	**11**
Sui (33)	5	Fair	1	1	1	1				1		1	1
Li et al. (35)	5	Fair	1	1		1			1			1	1
Geng et al. (29)	5	Fair	1	1		1				1		1	1
Zhang and Zhu (38)	7	Good	1	1	1	1			1	1		1	1
Wang (34)	6	Good	1	1		1				1	1	1	1
Zhao (32)	8	Good	1	1	1	1		1	1	1		1	1
Zheng (31)	6	Good	1	1	1	1				1		1	1
Zhu et al. (36)	7	Good	1	1	1	1			1	1		1	1
Fu et al. (37)	6	Good	1	1		1				1	1	1	1
Zhu (30)	5	Fair	1	1		1				1		1	1

*Studies were classified as having excellent (9–10), good (6–8), fair (4–5), or poor (<4)*.

*The PEDro scale involves (1) eligibility criteria; (2) random allocation; (3) concealed allocation; (4) similarity at baseline on key measures; (5) participant blinding; (6) instructor blinding; (7) assessor blinding; (8) more than 85% retention rate of at least one outcome; (9) intention-to-treat analysis; (10) between-group statistical comparison for at least one outcome; (11) point estimates and measures of variability provided for at least one outcome*.

### Synthesized results

**Figure 2:**
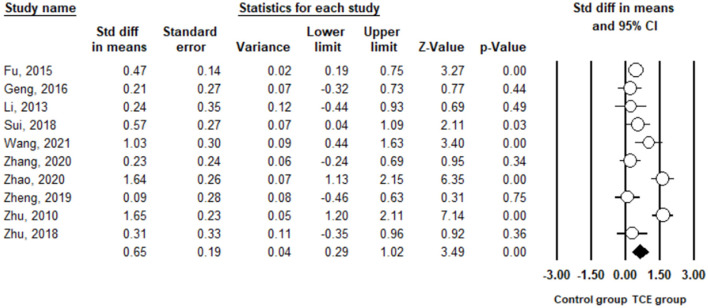
The effect of Traditional Chinese exercises on the depression.

**Figure 3:**
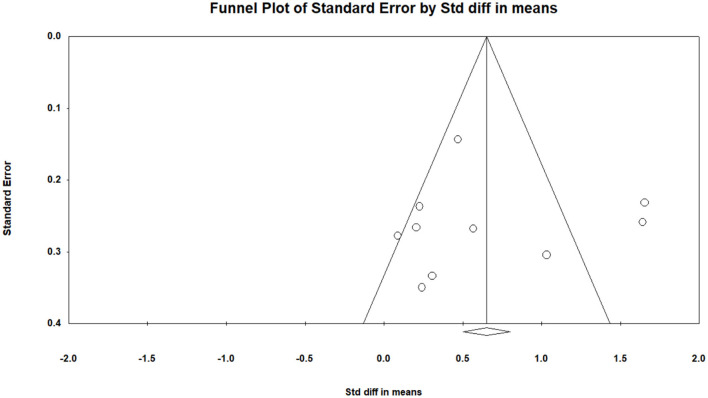
The funnel plot for publication bias in depression.

**Figure 4:**
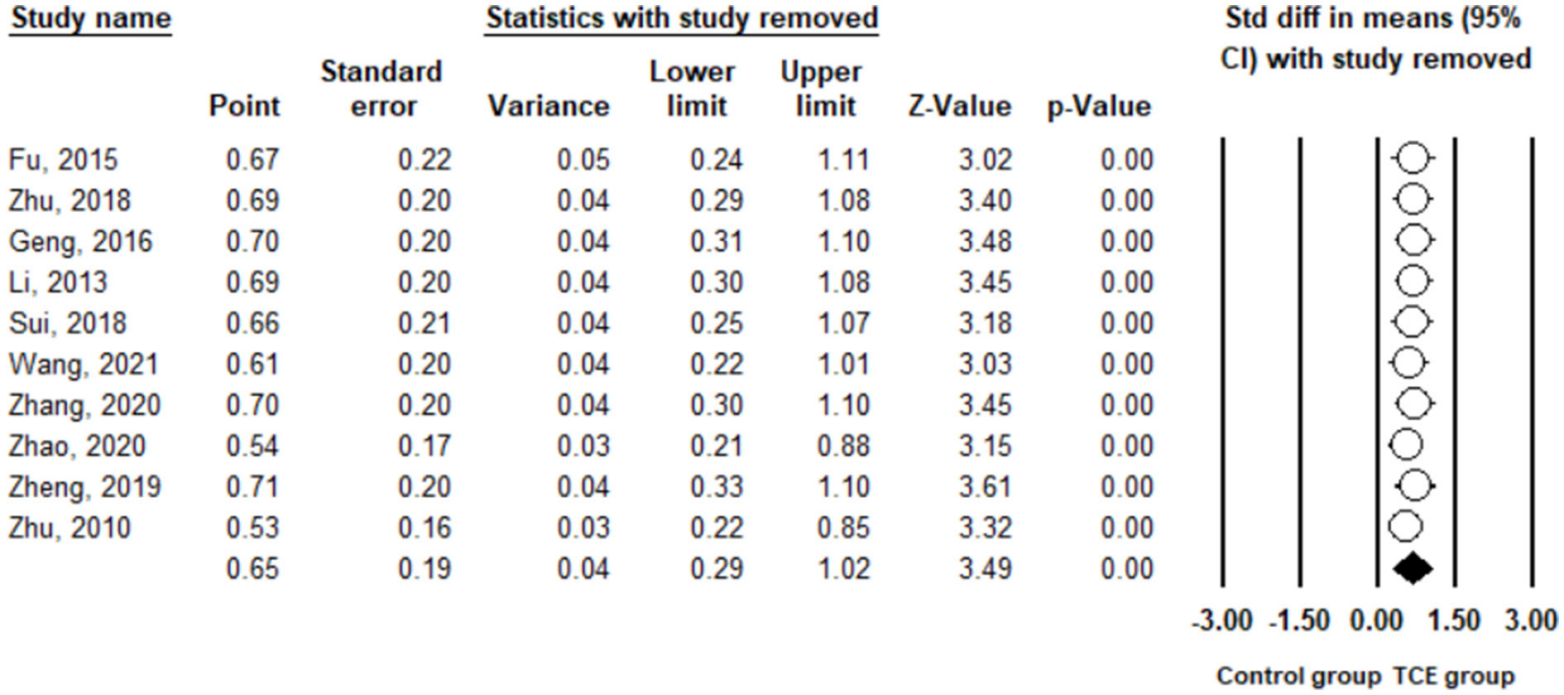
Leave one out sensitivity analysis for depression.

Depression was measured using SDS and BDI in the included studies. The pooled result from 10 studies (29–38) indicated that TCE had a positive effect on reducing depression compared to the control group (SMD = 0.65, 95% CI 0.29 to 1.02, I^2^ = 81.8%, *p* < 0.01) using a random-effects model (Figure 2). Publication bias was determined using the Egger's test (Egger's regression incept = 0.95, p > 0.05) and funnel plot (Figure 3). Furthermore, sensitivity analysis by removing one study at a time examined the sources of heterogeneity. The result revealed that the exclusion of any one study from the analysis did not influence the overall findings (Figure 4). Additionally, excluding two studies (30, 32) that produced smaller effect sizes to in the overall estimate did not change the result (SMD = 0.40, 95% CI 0.22 to 0.58, I^2^ = 7.7%, *p* < 0.01).

In the subgroup analysis (Table 3), when comparing active intervention (SMD = 0.33, 95% CI 0.07 to 0.60) with passive intervention (SMD = 1.07, 95% CI 0.40 to 1.74), significant improvements in depression were found in TCE. In particular, there was a greater magnitude of improvement in depression due to TCE compared to passive intervention. There was no significant difference in terms of sex. In other words, TCE showed a significant improvement for depression in both males (SMD = 0.84, 95% CI 0.16 to 1.52) and females (SMD = 0.46, 95% CI 0.21 to 0.71). With regard to the dose of exercise, significant differences in effect sizes in terms of exercise type (Q = 0.48, p = 0.49), exercise frequency (Q = 3.72, p = 0.16), exercise duration (Q = 3.20, p = 0.20), and exercise session time (Q = 0.03, p = 0.87) were not observed between categorical variables. However, practicing TCE for 3–4 times (SMD = 1.06, 95% CI 0.39 to 1.74) or 5 times or more (SMD = 0.39, 95% CI 0.12 to 0.66) per week showed a significant improvement in depression compared to 1–2 times (SMD = 0.24, 95% CI −0.44 to 0.93) per week. Practicing TCE for 12 weeks (SMD = 0.84, 95% CI 0.15 to 1.54) showed a significant improvement for depression compared to 13–23 weeks (SMD = 0.76, 95% CI −0.004 to 1.53) and 24 weeks or more (SMD = 0.25, 95% CI −0.08 to 0.58).

**Table 3:** Subgroup analysis for TCE versus control group on depression.

**Categorical**	**Level**	**No. of studies/**	**SMD**	**95% Confidence**	***I*^2^, %**	**Test for between-group**		
**moderator**		**comparisons**		**interval**		**hoterogeneity**		
						***Q*-value**	**df(*Q)***	***p*-value**
**Study design moderators**								
Control type	Active	6	0.33	0.07 to 0.60	24.2 %	4.02	1	0.04
	Passive	4	1.07	0.40 to 1.74	89.8%			
Sex	Male	5	0.84	0.16 to 1.52	89.2%	1.08	1	0.30
	Female	5	0.46	0.21 to 0.71	21.2%			
**Exercise moderators**								
Exercise type	TC	8	0.69	0.19 to 1.18	85.0%	0.48	1	0.49
	Qigong	2	0.49	0.24 to 0.74	0%			
Frequency	1–2	1	0.24	−0.44 to 0.93	0%			
	3–4	4	1.06	0.39 to 1.74	84.6%	3.72	2	0.16
	≥5	5	0.39	0.12 to 0.66	42.1%			
Exercise duration	12 weeks	4	0.84	0.15 to 1.54	85.6%			
	13–23 weeks	3	0.76	−0.004 to 1.53	89.5%	3.20	2	0.20
	≥24 weeks	3	0.25	−0.08 to 0.58	0%			
Exercise session time	≤ 45	5	0.68	0.10 to 1.27	82.8%	0.03	1	0.87
	>45	5	0.62	0.09 to 1.15	84.6%			

*SMD, standardized mean difference; df, degree of freedom*.

**Figure 5:**
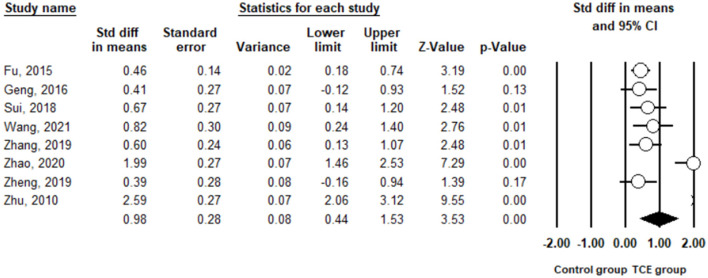
The effect of Traditional Chinese exercises on the anxiety.

**Figure 6:**
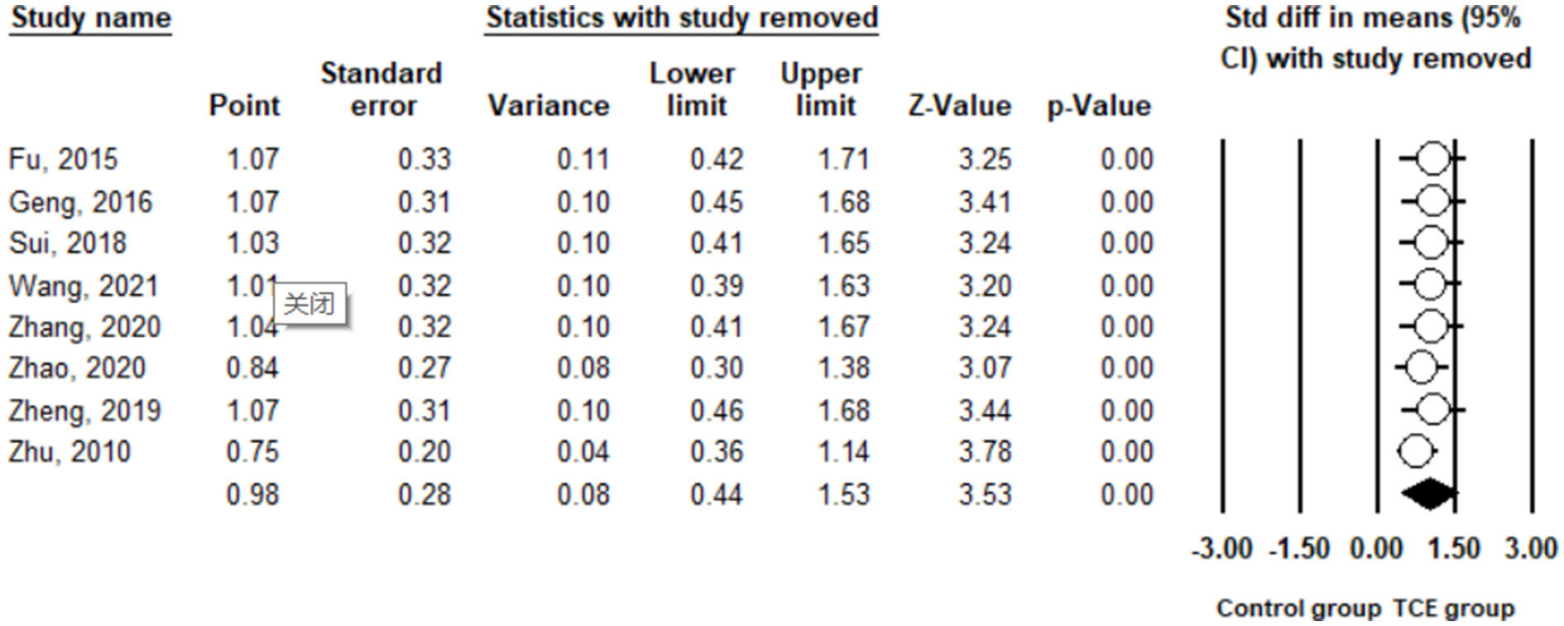
Leave one out sensitivity analysis for anxiety.

Anxiety was measured using the SAS in the included studies. The pooled result from eight studies (29–34, 37, 38) showed that TCE contributed to a significant reduction in anxiety compared to the control group (SMD = 0.98, 95% CI 0.44 to 1.53, I^2^ = 90.4%, *p* < 0.01) using a random-effects model (Figure 5). Furthermore, sensitivity analysis by removing one study at a time examined the sources of heterogeneity. The result revealed that the exclusion of any one study from the analysis did not influence the overall findings (Figure 6). Additionally, excluding two studies (30, 32) that produced smaller effect sizes on the overall estimate did not change the positive result (SMD = 0.52, 95% CI 0.34 to 0.71, I^2^ = 0%, *p* < 0.01).

**Figure 7:**
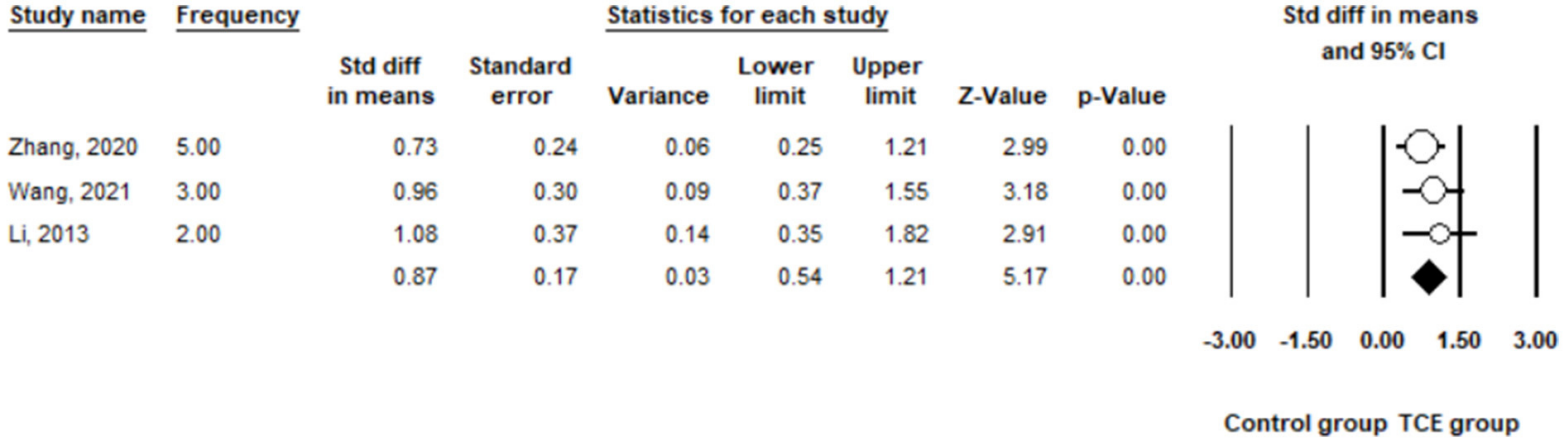
The effect of Traditional Chinese exercises on the drug cravings.

Drug cravings were measured in the term of psychological cravings in the three included studies (34, 35, 38). The pooled results indicated a significant reduction in DSD in the TCE group (SMD = 0.87, 95% CI 0.54 to 1.21, I^2^ = 0%, *p* < 0.01) when using a random-effects model (Figure 7).

**Figure 8:**
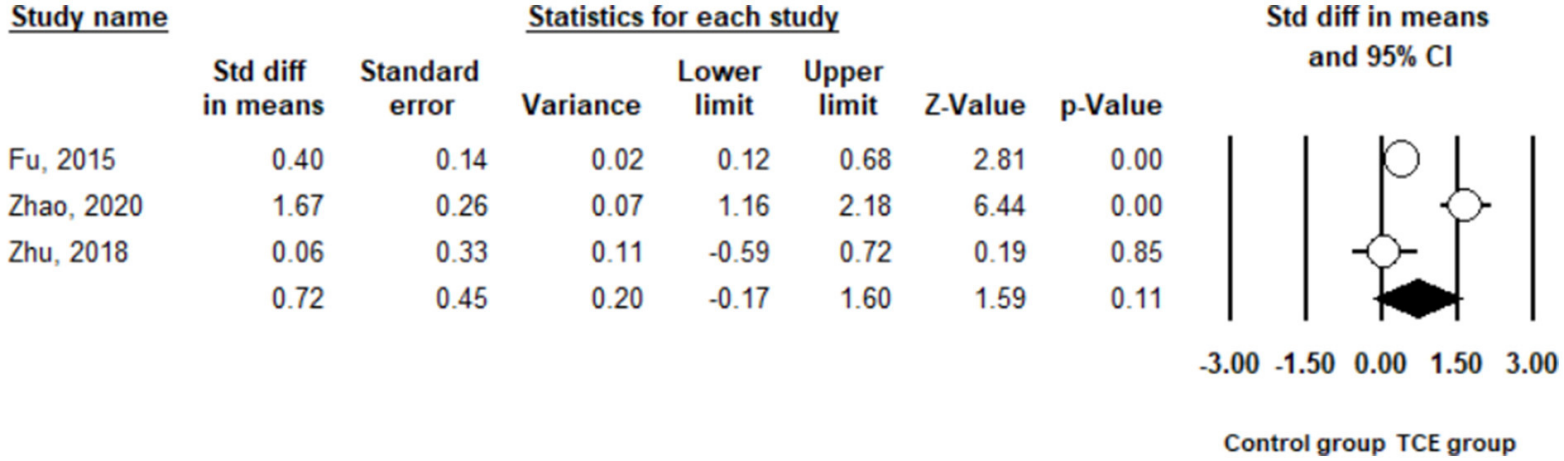
The effect of Traditional Chinese exercises on the sleep quality.

Sleep quality was measured using the PSQI in the three included studies (32, 36, 37). The pooled results indicated that there was no significant difference in terms of an improvement in the PSQI between the TCE and control groups (SMD = 0.72, 95% CI −0.17 to 1.60, I^2^ = 90.7%, *p* < 0.01) using a random-effects model (Figure 8).

## Discussion

The health benefits of TCE are increasingly being accepted by citizens and being used to manage mental health problems in drug rehabilitees. This systematic review and meta-analysis aimed to assess the existing studies about TCE (Tai Chi, Qigong) and mental health (depression, anxiety, and drug craving) among drug rehabilitee. The findings showed that TCE might be effective in reducing depression, anxiety and drug cravings among drug rehabilitees.

In this current study, some significant heterogeneities were observed in outcomes of depression and anxiety among the included studies, respectively. A sensitivity analysis was performed to examine the heterogeneity, accordingly. Zhao (32) and Zhu (30) reported larger effect sizes in favor of TCE. However, significant positive results were found after excluding these two studies, showing that TCE had significant effects in terms of reducing depression and anxiety in drug rehabilitees.

Exercise has been assumed to have beneficial effects on health outcomes in adults at different ages. The accumulation of exercise produces a series of positive statuses, reducing the mental health disorders (e.g., depression, anxiety, stress) of practitioners. In our study, the meta-analytic results found that TCE could significantly reduce depression and anxiety symptoms in drug rehabilitees. The ESs for depression (ES = 0.65) and anxiety (ES = 0.98) were moderate and larger, respectively. This implies that TCE has an effect on promoting people's mental health, assisting drug rehabilitees to reduce depression and anxiety symptoms. Our findings were consistent with previous studies (21, 40), which also found that mind-body exercises (e.g., Tai Chi, Yoga) could effectively relieve depression and anxiety symptoms of substance abuse users. Furthermore, according to the subgroup analysis of the present study, drug rehabilitees showed significant decreases in depression and anxiety symptoms after practicing Tai Chi and Qigong exercises. These findings were inconsistent with a previous meta-analysis study (22), which reported no significant effects in favor of Tai Chi exercises. Compared to the number of included studies (*n* = 3) in the previous study, our study enhanced the statistical power by including a greater number of studies (*n* = 8). Moreover, the studies included in our systematic review were randomized controlled trials, providing a more reliable result. Additionally, for the other doses of exercise, drug rehabilitee showed a significant decrease in depression after practicing TCE for 30 to 80 min, 3 times or more per week, for 12 weeks. This TCE exercise prescription is consistent with the WHO guidelines, which recommend that adults should perform more than 30 min of exercise at least 3 times per week. However, future research is needed to explore whether long-term TCE (more than 12 weeks) has a positive effect on depression in comparison with non-significant effect observed in our review study.

No previous review study has explored the role of sex on TCE and depression. Our subgroup analysis result also did not show a significant difference in male and female adults, and drug rehabilitees of both sexes could reduce their depression symptom by practicing TCE. The reason for this result is still unknown. It is likely that TCE like other exercise provided comprehensive benefits to mental health. Moreover, the different movements (e.g., meditation, breathing, and slow movements) of TCE could help people focus on the sensation of breathing, biofeedback, and immersive exercise. Thereby, both male and female practitioners strengthened their ability to promote relaxation and decrease depression episodes (41). Therefore, TCE can reduce depression symptom in male and female drug rehabilitees.

With respect to drug craving, previous studies have revealed that exercise has benefits in terms of reducing drug cravings in drug rehabilitee (34, 38). These results are in line with our finding, showing a significant decline in drug cravings with a larger effect (ES = 0.82) in favor of TCE in drug rehabilitees. In fact, long-term drug rehabilitees have a particularly strong dependence on drugs, which may be due to the decrease in dopamine secretion from the brain. Drug users can only take drugs to improve the unhappiness caused by a lack of dopamine, thus increasing their drug cravings. Moreover, in terms of the remarkable finding of the present study, it is likely that TCE contributes to a decrease in drug cravings by acting on other mediating factors, such as improved cognitive function. The key point of motivation for drug rehabilitees may be cravings, but some cognitive functions, such as memory and impulse control, may assist drug users in resisting these addictive impulses or cravings (42). A previous study indicated that engagement in exercise might enhance practitioners' self-cognitive ability to suppress drug cravings (43). Therefore, we believe that drug rehabilitee can reduce drug cravings after practicing TCE.

In addition, drug rehabilitees have an increased risk of sleep disorders and escape this ailment through drug abuse (44, 45). However, few experimental studies have examined the role of TCE in sleep disorders among drug rehabilitees. In our study, three eligible studies were included in the current meta-analysis, and they showed that TCE did not have a benefit of improving sleep quality among drug rehabilitees. This finding is inconsistent with previous studies that investigated the effect of TCE on sleep in people with or without diseases (46, 47). The results suggest a significant improvement in sleep quality in favor of TCE. The reason why TCE is able to assist in relieving sleep disorders is that TCE requires gentle and rhythmic movements; when practitioners perform TCE, the parasympathetic tone can be increased to regulate the nerve center and improve sleep quality. Moreover, TCE can bring pleasure to the body and mind of the practitioner and thereby reduce sleep disorders (48). However, the reason for the lack of a significant effect of TCE found in our study is that it is likely that our study focused on drug rehabilitees and a small number of studies were included compared with the population in the previous study. Thus, further research is needed to examine this finding.

There are several limitations that need to be acknowledged in this study. First, the participants included in the current study are mainly from China, where TCE is a popular exercise among people of different ages. This implies that more research needs to be completed that includes different ethnic groups in the future. Second, a limited number of studies were included and the studies were mainly conducted in China, which may cause a publication bias. Third, the heterogeneity across studies was relatively high due to the diversity of the study methodologies, such as different durations of intervention, different modalities of intervention, and different outcome measurements or test methods. Four, it is not known how long the TCE effect lasts after the intervention is discontinued, as the original study did not provide additional data to examine the results of the follow-up.

## Conclusion

The present systematic review and meta-analysis provides evidence that suggests that there are beneficial effects of TCE (Tai Chi, Qigong) in terms of relieving depression, anxiety, and drug cravings in drug rehabilitees. Further studies are warranted to verify our results by implementing well-designed experimental protocols.

## Data availability statement

The original contributions presented in the study are included in the article/supplementary material, further inquiries can be directed to the corresponding author/s.

## Author contributions

YZ and SL: conceptualization and methodology. YZ: data curation and analysis and writing—original draft preparation. SL: writing—review and editing. All authors have read and agreed to the published version of the manuscript.

## Funding

This study was supported by the Hunan Judicial Police Vocational College, and the Philosophy and Social Science Planning Project in Hunan Province (Grant Number: 19YBQ082).

## Conflict of interest

The authors declare that the research was conducted in the absence of any commercial or financial relationships that could be construed as a potential conflict of interest.

## Publisher's note

All claims expressed in this article are solely those of the authors and do not necessarily represent those of their affiliated organizations, or those of the publisher, the editors and the reviewers. Any product that may be evaluated in this article, or claim that may be made by its manufacturer, is not guaranteed or endorsed by the publisher.
